# Supplementary material for: Loss of iron triggers PINK1/Parkin-independent mitophagy
Source: EMBO Rep. 2013 Nov 1;14(12):1127–35. doi: 10.1038/embor.2013.168 (PMC3981094; doi:10.1038/embor.2013.168)
Supplement: Supplementary Information [file embor2013168s1.pdf]

Supplemental Information

Supplemental Methods

Materials

Bafilomycin A1 and Dimethyloxaloylglycine (DMOG) were purchased from Ezno Life Sciences (Exeter, UK); Rapamycin, Bortezomib and pyridoxal isonicotinoyl hydrazone (PIH) were purchased from Merck-Millipore (Darmstadt, Germany); KU-0063794 and A-769662 were purchased from Tocris Biosciences (Bristol, UK), the specificity of KU-0063794 as an mTOR inhibitor has been previously determined [1] and it is a recommended autophagy agonist [2]; MitoTracker Red FM, MitoTracker Deep Red FM, LysoTracker Deep Red, Lipofectamine RNAiMAX, Lipofectamine 2000, Earl's Balanced Salt Solution (EBSS), Dulbecco's Modified Eagle Medium (DMEM) with and without glucose and Ham's F-12 nutrient mix were purchased from Life Technologies (Paisley, UK). Foetal Bovine Serum (FBS) was purchased from Thermo Scientific (Loughborough, UK). Unless otherwise stated all other reagents were purchased from Sigma-Aldrich (Poole, UK) or VWR International (Lutterworth, UK).

Cell Treatments

For experiments cells were treated for 24h with a final concentration of 1mM DFP, except where stated, dissolved in H_2O at +95°C and sterile filtered. The following chemicals were added to cell media at final concentrations: 50nM or 100nM Bafilomycin A1, 20µM CCCP, 500nM Rotenone, 1µM Antimycin A, 20µM FCCP, 100µM 2,4-dinitrophenol (DNP), 10µM Oligomycin A, 10µM valinomycin, 20µM citrinin, 10µM A-769662, 1µM KU0063794, 100nM Rapamycin, 1mM DMOG, 25µM Dp44mT, 100µM pyridoxal isonicotinoyl hydrazine (PIH), 1µM Bortezomib each made up in DMSO; 1mM 2,2'-bipyridyl was made up in Ethanol; 1mM DFO, 7µM sodium selenite, 500µM L-Buthionine sulfoxamine (L-BSO), 5mM nicotinamide, 100µM $CoCl_2$, 5mM N-acetyl L-cysteine (NAC) each made up in H_2O. For amino acid starvation cells were washed twice in EBSS and then incubated in EBSS for the indicated length of time. For hypoxia treatment cells were transferred to a Ruskinn INVIVO$_2$ 300 hypoxia hood (Ruskinn, Bidgend, UK) at +37.0°C in 0.5% O_2, 5.0% CO_2 for 24h. For lysis cells were washed twice with PBS on ice and subsequently scraped into ice cold lysis buffer (50mM HEPES pH7.4, 150mM NaCl, 1mM EDTA, 10% glycerol, 0.5% NP-40, 1mM DTT, 1mM PMSF, and phosphatase inhibitors), incubated on ice for 10min and centrifuged at 20000xg for 10min at +4°C. Supernatants were retained for analysis and protein concentration measured using Bio-Rad protein assay (Bio-Rad, Hemel Hempstead, UK).

RNA interference

The shRNA construct against human BNIP3 was cloned into the pSuperRetro.Puro vector according to manufacturer's instructions (Oligoengine, Seattle, USA) and cells transduced with retroviral particles as described for the tandem tagged mitochondrial construct. The BNIP3 RNAi sequence used was GGAAAGAAGTTGAAAGCAT. Custom siRNAs against

ATG5 and Beclin1 were purchased from Thermo Scientific, ATG5: GGAAUAUCCUGCAGAAGAAUU and Beclin1: GCUCAGUAUCAGAGAAAUUU. siRNAs were transfected using Lipofectamine RNAiMAX (Life Technologies) in accordance with manufacturer's instructions. Validated siRNA against PINK1 was purchased from Sigma-Aldrich (Product No. SASI_Hs01_00022856) and transfected using Transfectin (Bio-Rad) in accordance with manufacturers instructions. Non-targeting control siRNA was purchased from Thermo Scientific (Product No. D-001810-10-20).

Immunoblotting

20-30μg of cell lysates were resolved by SDS-PAGE and transferred to PVDF. Primary antibodies were incubated in Tris-buffered saline with 0.1% Tween20 (TBST) with 5% bovine serum albumin (BSA) for 16h at +4°C. The following commercial primary antibodies were used: from Cell Signalling Technology (Danvers, USA) LC3A/B (4108, 1:1000), BNIP3 (3769, 1:1000), HSP60 (4870, 1:1000) PDH subunit E1α (2784, 1:1000); from MBL (Woburn, USA) Beclin 1 (PD017, 1:5000); from Abcam (Cambridge, UK) Mitofusin 2 (ab56889, 1:1000), Timm50 (ab23938, 1:1000); from Abnova (Taipei, Taiwan) p62/SQSTM1 (H00008878-M01, 1:10,000); from R&D systems (Minneapolis, USA) HIF-1α (MAB1536, 1:1000); from Santa Cruz Biotechnology (Dallas, USA) Parkin (sc-32282, 1:1000); from Novus Biologicals (Cambridge, UK) PINK1 (BC100-494, 1:2000); from Sigma-Aldrich ATG5 (A0731, 1:1000); from Life Technologies Transferrin Receptor (13-6800, 1:2000); from Merck-Millipore α-tubulin (CP06, 1:5000). Primary Omi antibody was produced in-house in sheep raised against GST-Omi. Membranes were washed and subsequently incubated with HRP-conjugated secondary antibodies (Thermo Scientific) for 1h at RT. Membranes were developed using ECL (GE Healthcare, Amersham, UK) and exposed to X-ray film. X-ray film was scanned using an Epson perfection V700 photo scanner with Epson Scan 3.28E software (Epson, Hemel Hempstead, UK). Western blotting data was quantified using Image J software (http://rsbweb.nih.gov) and all data was normalised to the loading control α-tubulin.

Immunofluorescence and Fixed Cell Mitotracker Staining

Cells were grown on glass coverslips and cultured/treated as described above. For mitotracker staining cells were incubated in culture medium containing 200nM mitotracker Red FM for 30min and washed twice with PBS prior to fixation. Cells were fixed with 3.7% formaldehyde, 50mM HEPES pH7.4 for 20min. Formaldehyde was quenched with DMEM, 10mM HEPES pH7.4. Cells were permeablised with 0.2% NP-40 in PBS for 3min, blocked for 15min with 1% BSA in PBS (blocking buffer), incubated with primary antibody in blocking buffer for 1h at +37˚C, washed 3 x 10min in blocking buffer, incubated with Alexafluor coupled secondary antibodies (Life Technologies) in blocking buffer for 30min and washed 3 x 10min in blocking buffer. Cells were mounted using prolong gold mounting solution with dapi (Life Technologies) and visualised with a Nikon Eclipse Ti-S fluorescence microscope (Nikon, Kingston Upon Thames, UK). Primary antibodies were ATP synthase subunit beta (Abcam, ab13740, 1:500), LC3 (MBL, M152, 1:500), COXIV (Cell Signalling Technology, 4850, 1:500) and PINK1 (Novus Biologicals, BC100-494, 1:500). Images were

quantified by manual counting of three fields of view using Nikon NIS-Elements software and images processed using Adobe Photoshop CS5.1 (Adobe, San Jose, USA).

Live Cell Lysotracker and Mitotracker Staining

Cells were grown on glass bottomed dishes and cultured/treated as described above. For lysotracker staining cells were incubated in culture medium containing 50nM LysoTracker Deep Red for 1 hour and for mitotracker staining cells were incubated in culture medium containing 200nM MitoTracker Deep Red FM for 30min. Cells were washed once with PBS and then incubated at +37˚C in DMEM, 10mM HEPES, without phenol red and images taken of live cells using a Nikon Eclipse TE200 microscope and Applied Precision softWoRx 5.5 software (GE Healthcare). Images were processed using Adobe Photoshop CS5.1.

Citrate Synthase Activity

Citrate synthase activity was determined by the method of [3]. Cell lysate was incubated with 100mM Tris pH8.0, 0.1% triton X-100, 0.1mM acetyl-coenzyme A and 0.2mM 5'-dithio-bis (2-nitrobenzoic acid). Reaction was started with 0.2mM oxaloacetate and the reaction was measured at 405nm for 1.5min at 10sec intervals at +30˚C using a VersaMAX plate reader (Molecular Devices, Sunnyvale, USA).

Electron Microscopy

Cells were washed twice with PBS and subsequently lifted with 0.48mM EDTA in PBS. Cells were collected by centrifugation at 100xg for 2min. Cells were fixed with 2.5% glutaraldehyde, 2% formaldehyde in 0.1M sodium cacodylate. Fixed cells were dehydrated in graded ethanol and propylene oxide and embedded in durcupan resin (Sigma-Aldrich). Sections were cut on a Leica UCT ultramicrotome (Leica Microsystems, Milton Keynes, UK), collected on Pioloform coated 100 mesh copper grids and stained with uranyl acetate and lead citrate. Sections were examined on a Jeol 1200 EX electron microscope (Jeol, Welwyn Garden City, UK) and images taken on Fuji digital imaging plates and processed in a Ditabis plate scanner (Ditabis, Pforzheim, Germany). Images were quantified by manual counting of single or double membrane structures containing mitochondria. 50 cell sections (cross sections through 50 cells) were counted per condition for each independent experiment.

Oxygen Consumption

Oxygen consumption was measured using a Seahorse XF24 Extracellular Flux analyser (Seahorse Bioscience, North Billerica, USA). Cells were cultured in 24 well Seahorse plates at a density of $8x10^4$ cells/well and incubated with DFP for the indicated length of time. Cell culture medium was replaced with unbuffered DMEM containing 200mM GlutaMax-1, 100mM Na pyruvate, 25mM glucose, 32mM NaCl and 40µM Phenol Red. Oxygen consumption was measured and respiration rate analysed with injections of 1µM oligomycin, 1µM FCCP and 10µM antimycin A as previously described for SH-SY5Y cells [4]. Results were normalised to total protein determined using the BCA assay (Thermo Scientific)

ATP/ADP Ratios

Cells were washed once in ice cold PBS, lysed in 300µL 5% percholric acid, vortexed and centrifuged at 17700xg for 3min. Supernatent was extracted with 3 washes of a 1:1 mixture of tri-n-octylamine and 1,1,2-trichlorotriflouroethane. The aqueous phase was separated by capillary electrophoresis and nucleotides detected by UV absorbance at 260nm as previously described [5].

Galactose Incubation

All cells were washed twice with glucose-free DMEM with 10mM galactose + 10% FBS. Cells grown under glucose were then incubated in DMEM containing 25mM glucose + 10% FBS and cells grown under galactose were incubated in glucose-free DMEM containing 10mM galactose + 10% FBS for 48 hours prior and during treatment with 1mM DFP as described above.

Reactive Oxygen Species Detection

Cells were cultured in 96 well plates at a density of $2.5x10^4$ cells/well and treated with DFP as indicated. 5mM N-acetylcysteine was added at the same time as DFP or vehicle control. Following incubations cells were washed once with PBS and incubated for 30min at +37˚C in DMEM with 1% FBS containing 100µM 2',7'-dichlorofluorescin diacetate (DCFDA). Cells were washed once with Krebs-Ringer-HEPES (KRH) buffer and then 200µL KRH buffer was added to each well. 0.1% H_2O_2 was added where indicated and fluorescence measured at excitation wavelength 485nm and emission wavelength 538nm using a SpectraMAX Gemini EM plate reader (Molecular Devices). Results were normalised to the amount of cell material which was measured using the sulforhodamine B assay performed as previously described [6].

Supplemental references

1. Garcia-Martinez, J.M., et al. (2009) Ku-0063794 is a specific inhibitor of the mammalian target of rapamycin (mTOR). The Biochemical journal 421: 29-42
2. Klionsky, D.J., et al. (2012) Guidelines for the use and interpretation of assays for monitoring autophagy. Autophagy 8: 445-544
3. Shepherd, D. and P.B. Garland (1969) The kinetic properties of citrate synthase from rat liver mitochondria. The Biochemical journal 114: 597-610
4. Schneider, L., et al. (2011) Differentiation of SH-SY5Y cells to a neuronal phenotype changes cellular bioenergetics and the response to oxidative stress. Free radical biology & medicine 51: 2007-17
5. Hawley, S.A., et al. (2010) Use of cells expressing gamma subunit variants to identify diverse mechanisms of AMPK activation. Cell metabolism 11: 554-65
6. Vichai, V. and K. Kirtikara (2006) Sulforhodamine B colorimetric assay for cytotoxicity screening. Nature protocols 1: 1112-6

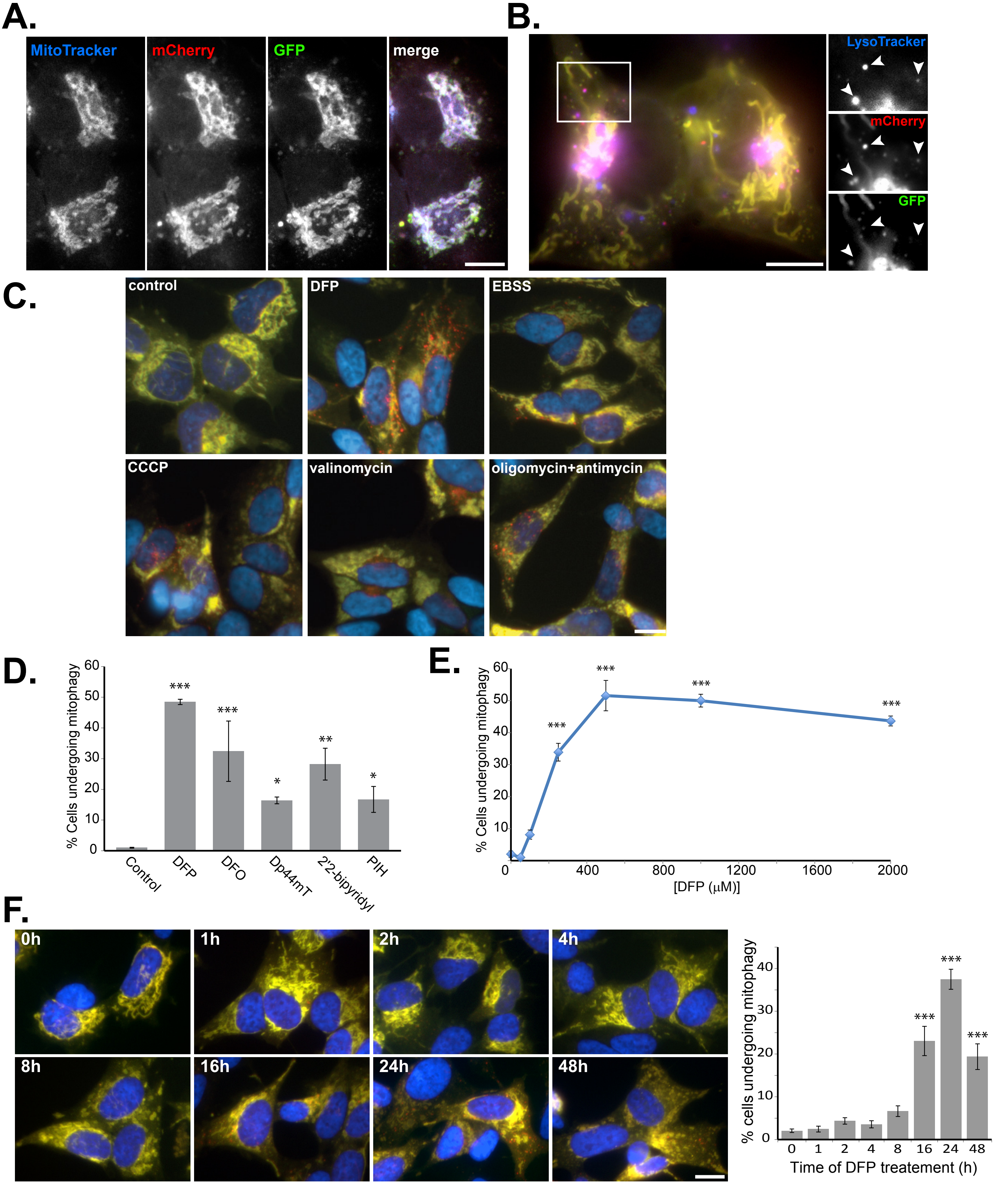

Figure S1. Tandem tag mitophagy assay. A. Co-localisation of mCherry-GFP-FIS1101-152 expressed in SH-SY5Y cells with Mitotracker Deep Red. B. Co-localisation of red-alone puncta with Lysotracker Deep Red in SH-SY5Y cells expressing mCherry-GFP-FIS1101-152 treated with 1mM Deferiprone (DFP) for 24h. C. Representative micrographs of conditions that induced a significant increase in mitophagy (see fig 1C for quantitation). All treatments for 24h, EBSS (Earl's Balanced Salt Solution) is amino acid starvation. D. Results of tandem tag mitophagy assay in SH-SY5Y cells treated for 24h with the following iron chelators: 1mM DFP, 1mM Deferoxamine (DFO), 25μM Dp44mT, 1mM 2'2-bipyridyl, 100μM pyridoxal isonicotinoyl hydrazone (PIH). E. Results of tandem tag mitophagy assay in SH-SY5Y cells treated with DFP at a range of concentrations for 24h. F. Representative images and results of tandem tag mitophagy assay in SH-SY5Y cells treated with 1mM DFP over a time course. All results are from 3 independent experiments. All quantitative data are mean ± SEM. *P<0.05, **P<0.01, ***P<0.001, N.S. not significant. Scale bars: 10μm.

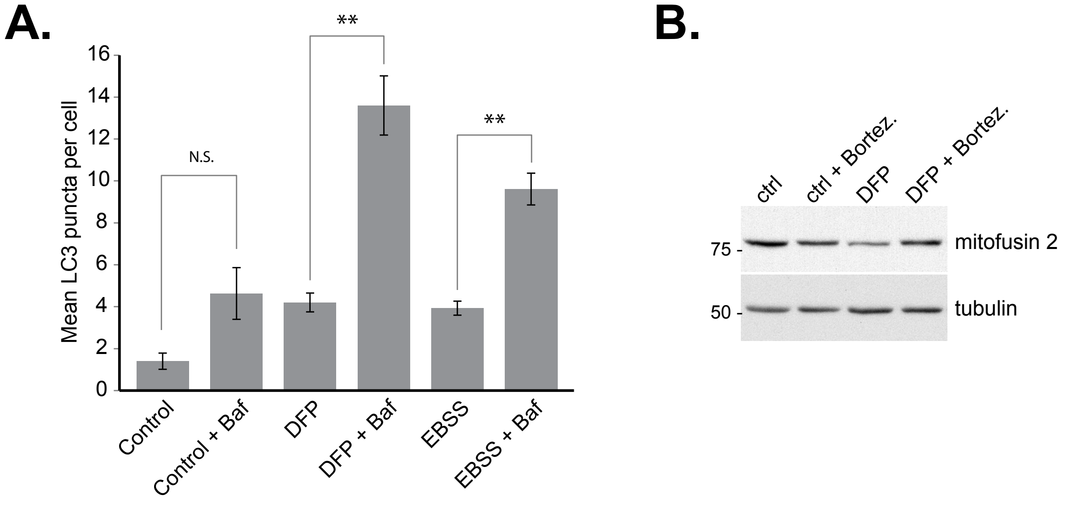

Figure S2. DFP induces autophagy and proteosomal degradation of mitofusin 2. A. Quantitation of immunofluorescence images of LC3 positive autophagic puncta in SH-SY5Y cells stained with antibodies against autophagic marker LC3 and mitochondrial complex IV component COXIV. Cells were treated with 1mM Deferiprone (DFP) for 16h, EBSS (amino acid starvation) for 2h and where indicated 50nM Bafilomycin A1 (Baf) for the final 2h of treatment. This is quantified data from the same experiment presented in Figure 2A and 2B. B. Representative immunoblot of mitofusin 2 (MFN2) in SH-SY5Y cells treated with 1mM DFP and/or the proteasomal inhibitor Bortezomib (Bortez.) at 1μM for 24h. All results are from 3 independent experiments. All quantitative data are mean ± SEM. **P<0.01, N.S. not significant.

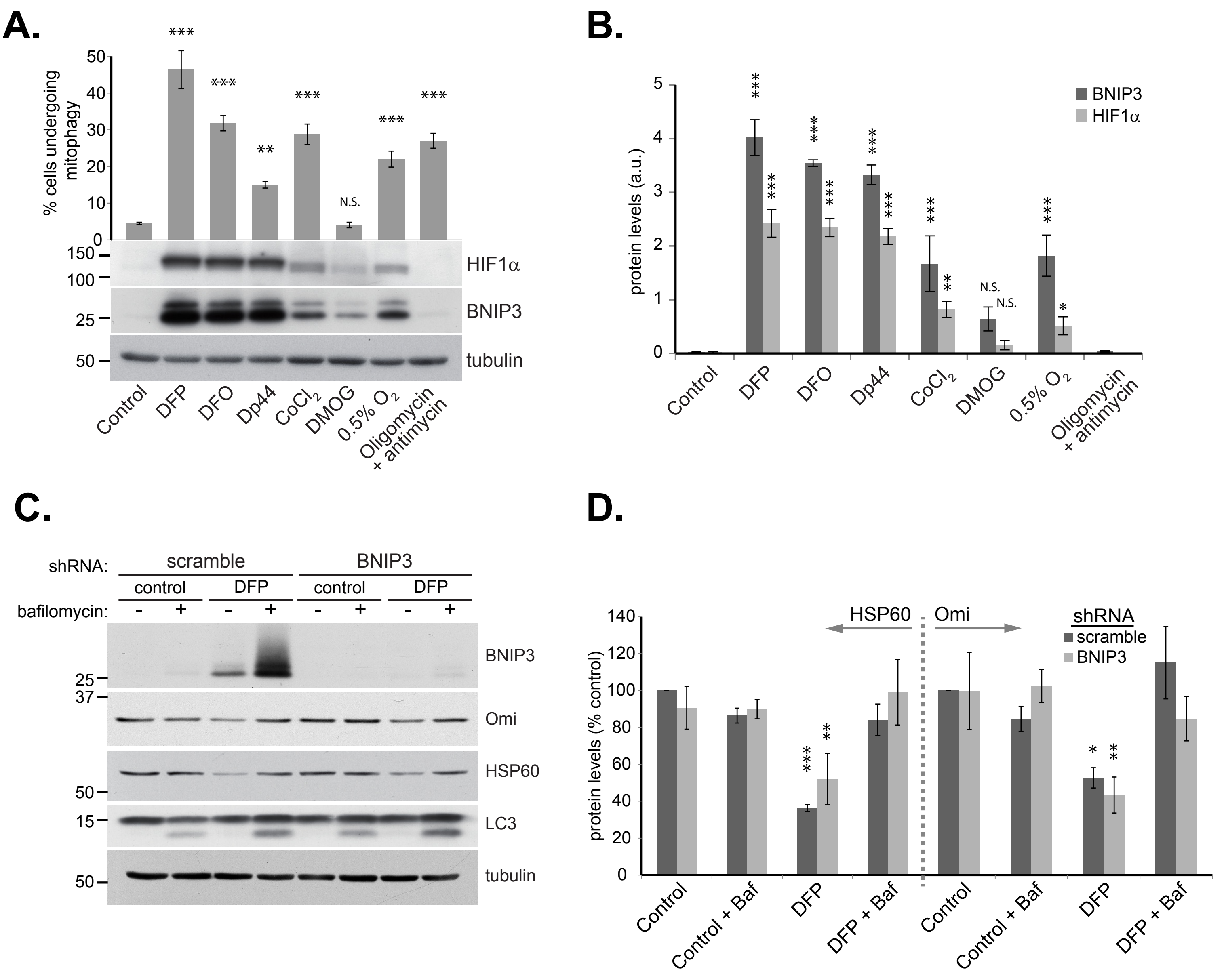

Figure S3. HIF1α stabilisation and mitophagy. A. Induction of mitophagy by conditions that lead to the stabilisation of HIF1α in SH-SY5Y cells using the tandem tag mitophagy assay. Conditions previously known to stabilise HIF1α were used including iron chelation (Deferiprone (DFP), Deferoxamine (DFO) and Dp44mT), CoCl2, Dimethyloxalylglycine (DMOG) and hypoxia (0.5% O2). Representative blots in A. and quantitation in B. demonstrating HIF-1α stabilisation and induction of BNIP3 (a HIF1 responsive gene) in cells treated in the same experiment as mitophagy measurements in A. C. Representative blot and D. Quantitation of mitochondrial proteins Omi and HSP60 and autophagy marker LC3 in SH-SY5Y cells depleted of BNIP3 with shRNA and treated with 1mM DFP for 24h. Where indicated 50nM Bafilomycin A1 was added for the final 16h of treatment. All results are from 3 independent experiments. All quantitative data are mean ± SEM. *P<0.05, **P<0.01, ***P<0.001, N.S. not significant.

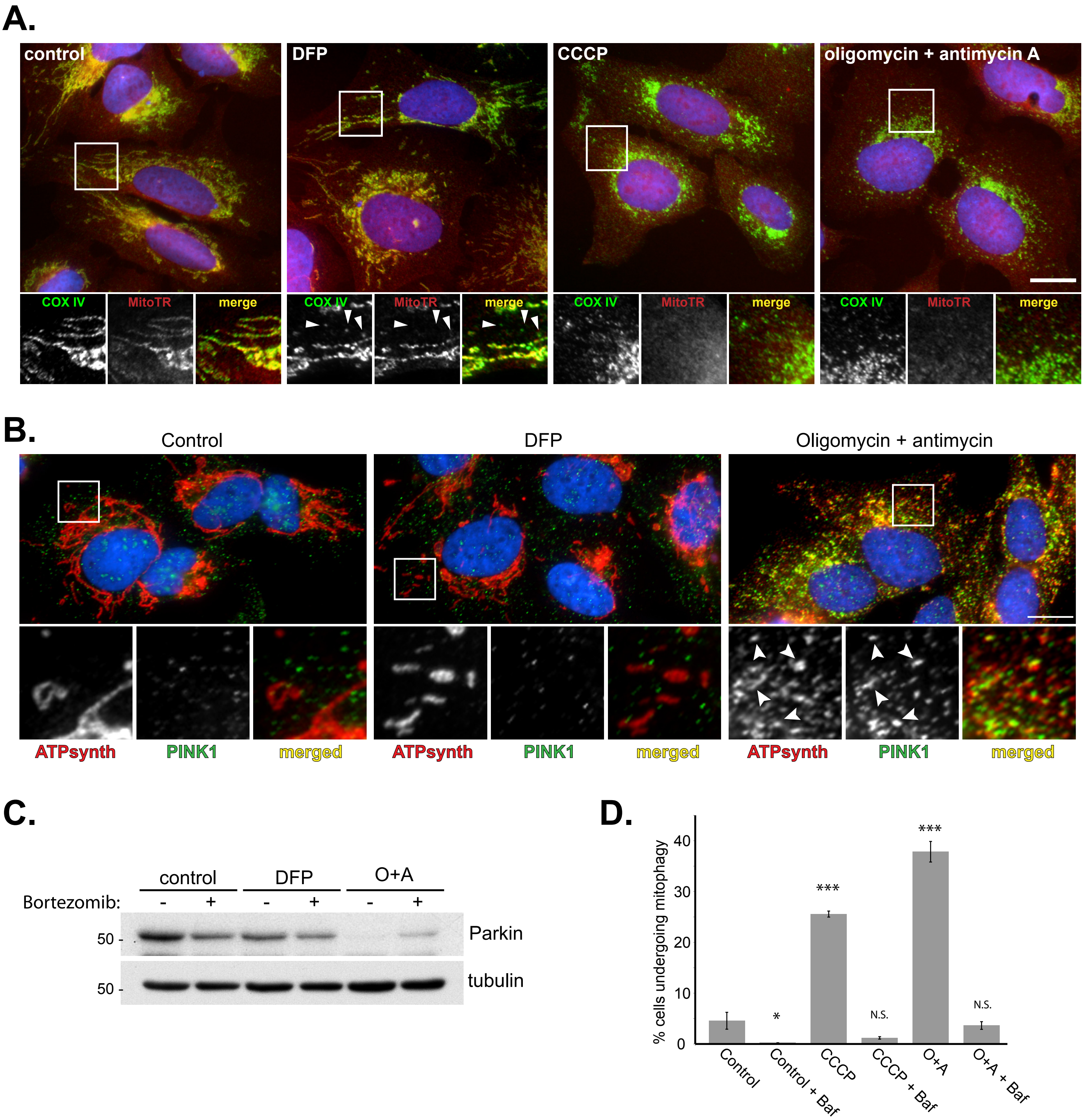

Figure S4. Comparison of DFP treatment to Oligomycin A + Antimycin A and CCCP treatments.
A. Representative mitochondrial complex IV component COXIV immunostaining and mitotracker co-staining of SH-SY5Y cells treated with 1mM Deferiprone (DFP), 20μM CCCP or 10μM Oligomycin A + 1μM Antimycin A for 24h. Arrowheads indicate COXIV positive punctate structures that are negative for mitotracker and could represent depolorised mitochondria in autophagosomes or lysosomes. B. Representative Mitochondrial ATP synthase subunit beta (ATP synth) and PINK1 immunostaining of SH-SY5Y cells following treatment with 1mM DFP or 10μM Oligomycin A + 1μM Antimycin A for 24h. C. Representative immunoblot of Parkin in SH-SY5Y cells treated with 1mM DFP or 10μM Oligomycin A + 1μM Antimycin A for 24h. Where indicated the proteasomal inhibitor Bortezomib (Bortez.) at 1μM was added for 24h. D. Results of tandem tag mitophagy assay in SH-SY5Y cells following treatment with 20μM CCCP or 10μM Oligomycin A + 1μM Antimycin A for 24h. Where indicated 100nM Bafilomycin A1 (Baf) was added for final 4h of incubation. All results are from 3 independent experiments. All quantitative data are mean ± SEM. *P<0.05, ***P<0.001, N.S. not significant. Scale bars: 10μm.
