# Supplementary figures and images for: Loss of iron triggers PINK1/Parkin-independent mitophagy
Source: EMBO Rep. 2013 Nov 1;14(12):1127–35. doi: 10.1038/embor.2013.168 (PMC3981094; doi:10.1038/embor.2013.168)

Figure S2B.

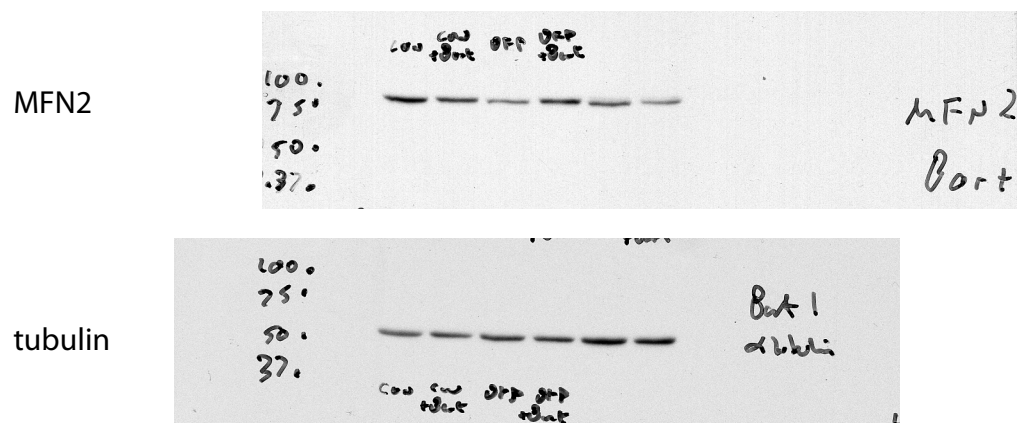

Supplement: Supplementary Information [file embor2013168dfs2.pdf]

Figure S3A.

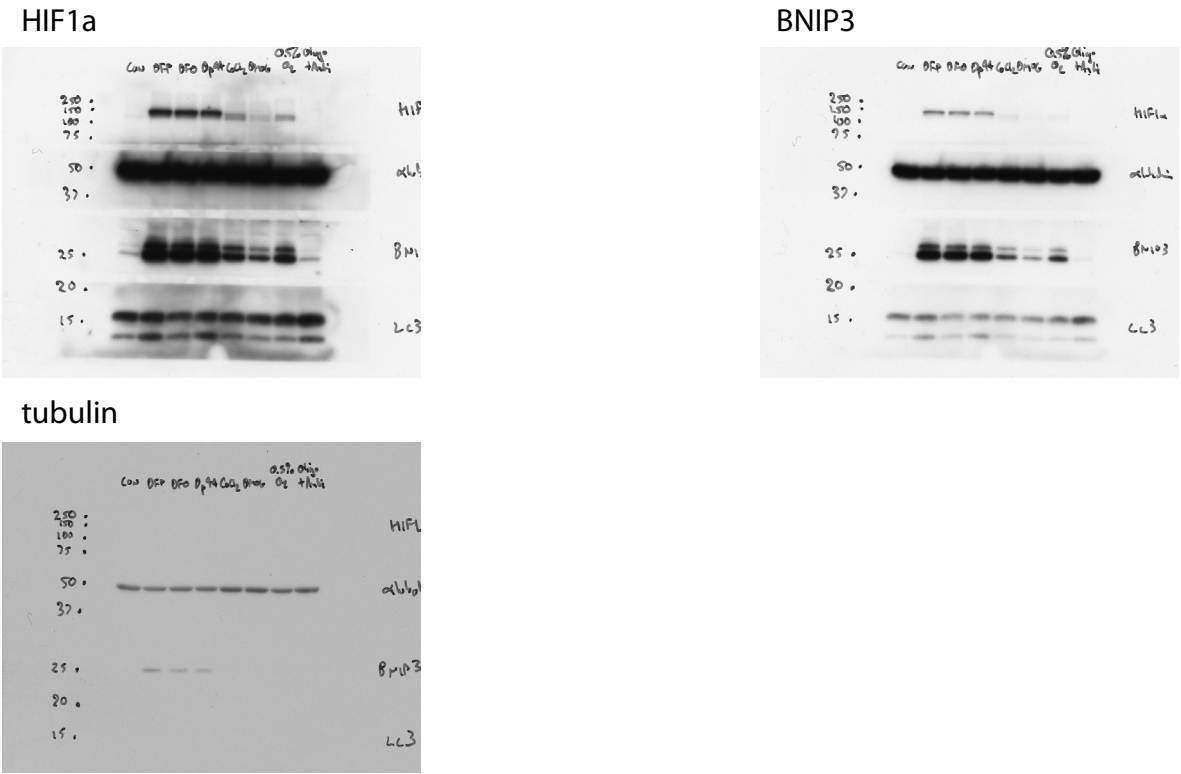

Figure S3C.

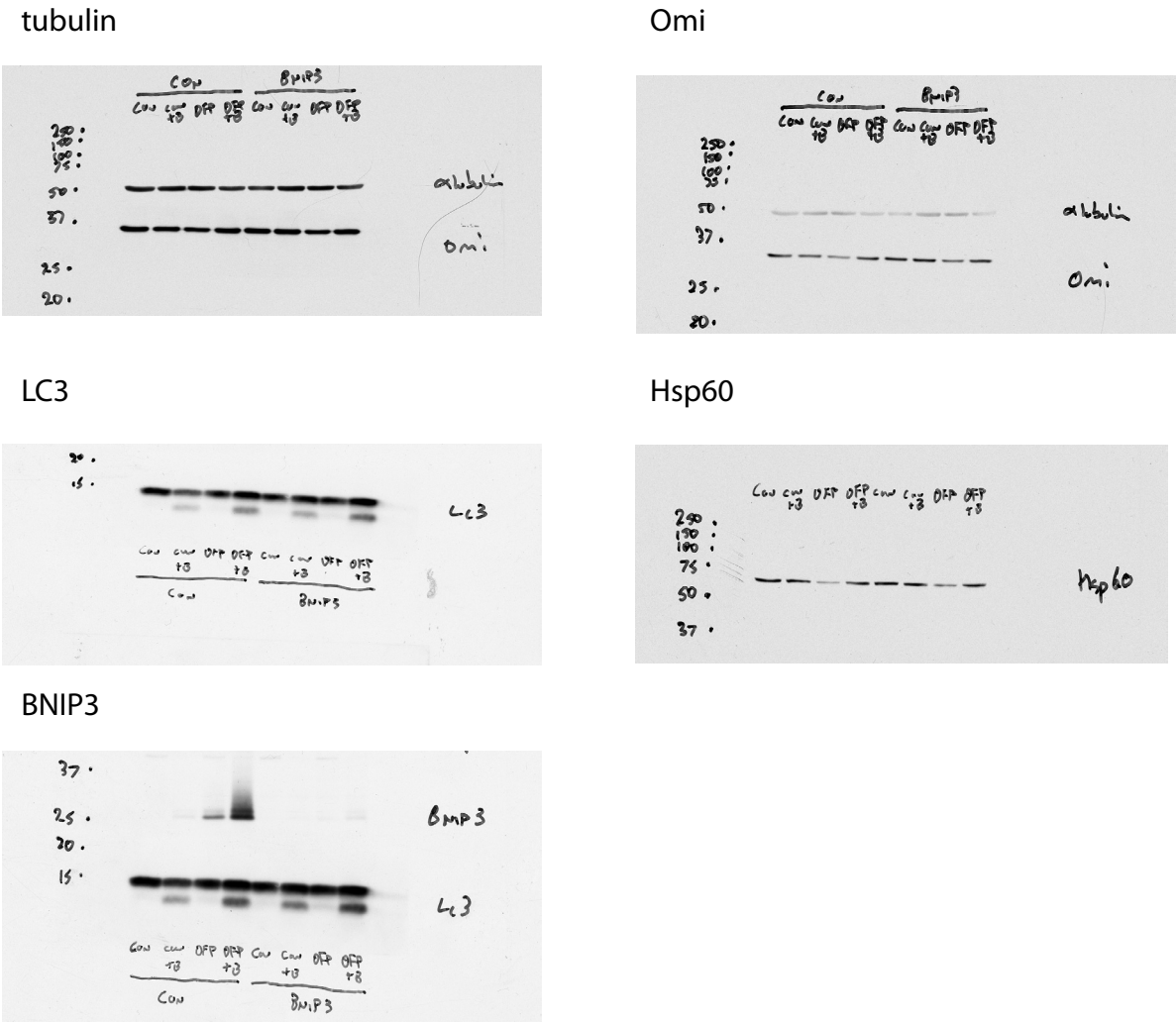

Supplement: Supplementary Information [file embor2013168dfs3.pdf]

Figure S4C.

Parkin

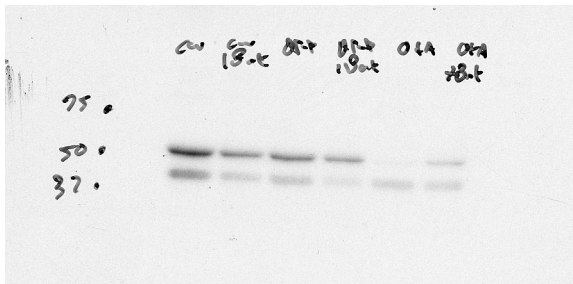

tubulin

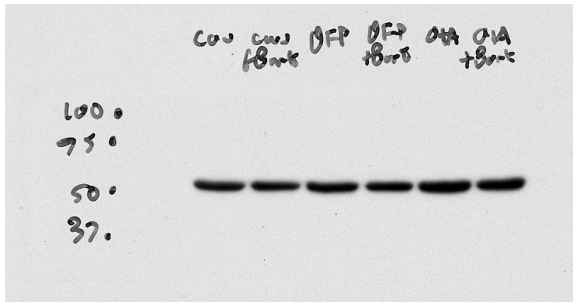

Supplement: Supplementary Information [file embor2013168dfs4.pdf]

Figure1F.

Transferrin Receptor

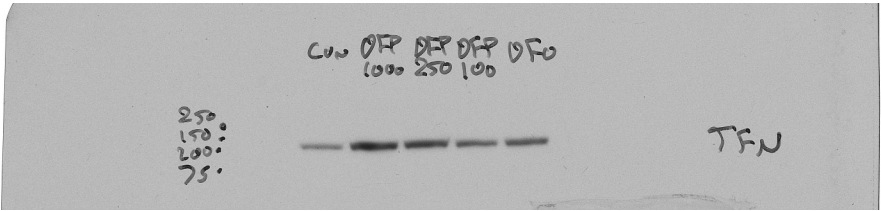

tubulin

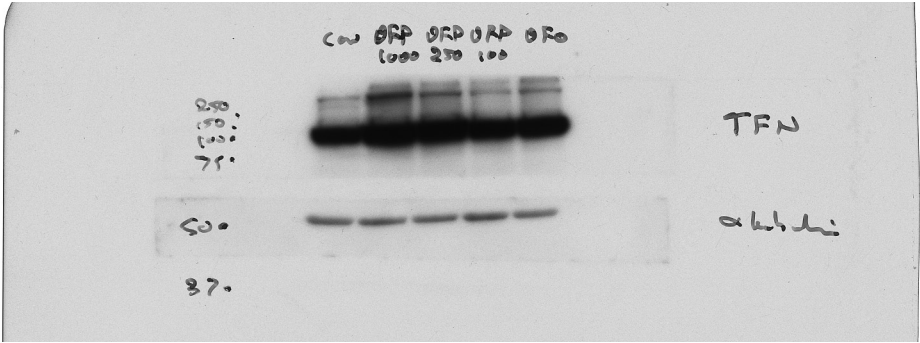

Supplement: Source data for Figure 1 [file embor2013168df1.pdf]

Figure 2C.

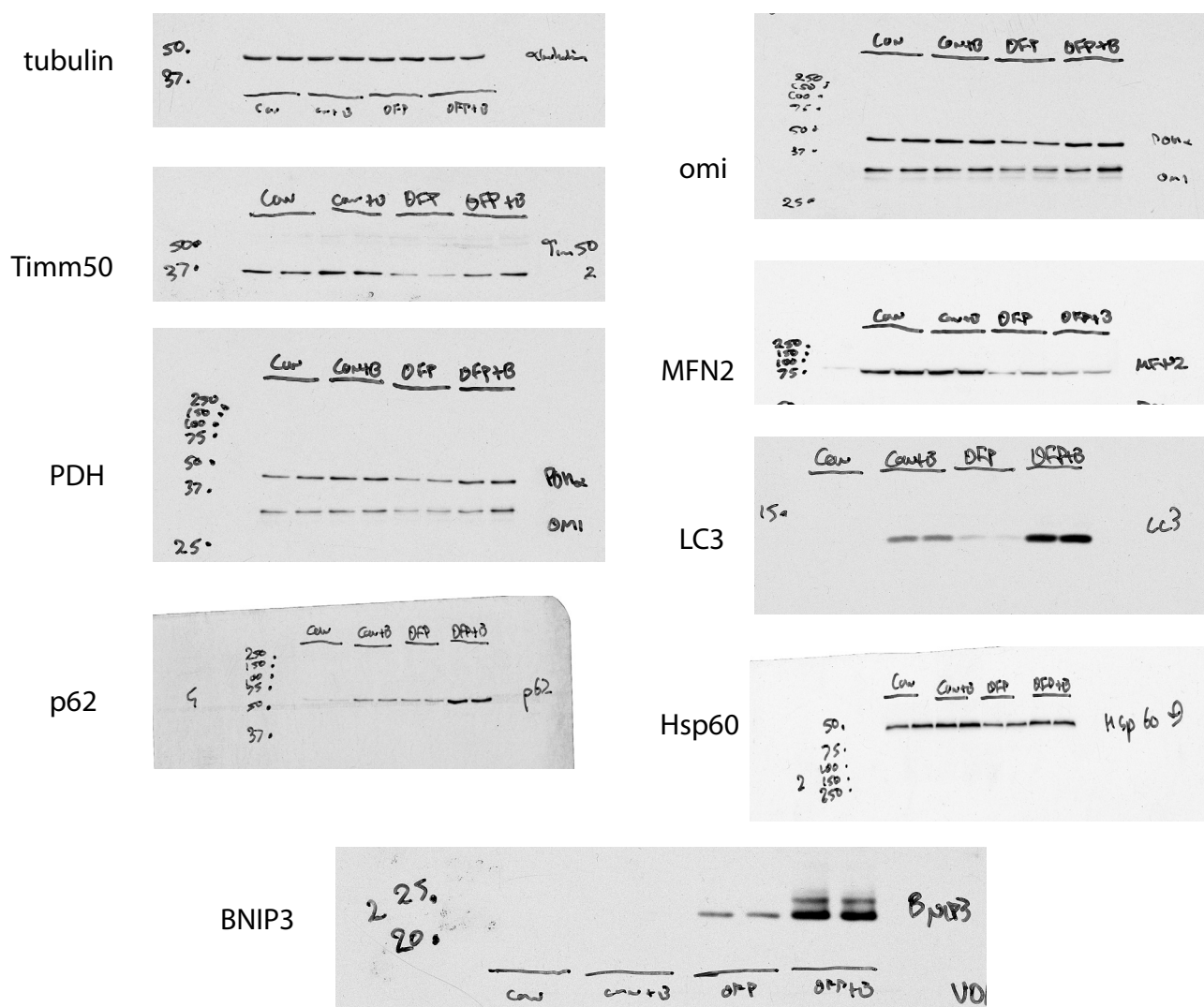

Figure 2F.

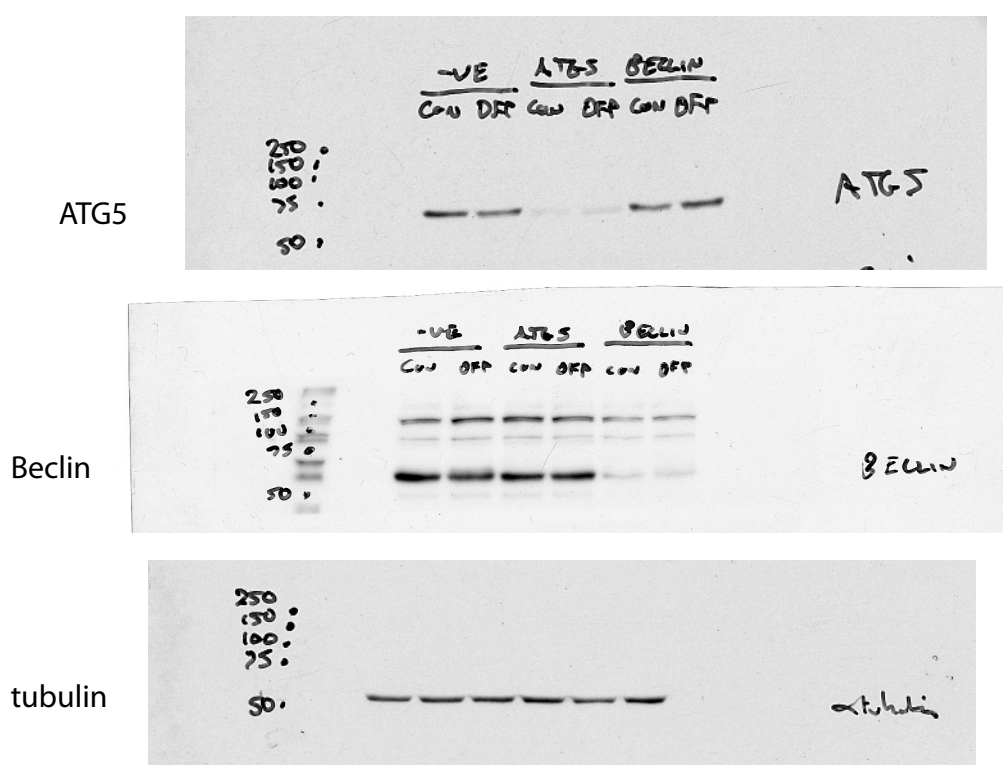

Supplement: Source data for Figure 2 [file embor2013168df2.pdf]

Figure 4A.

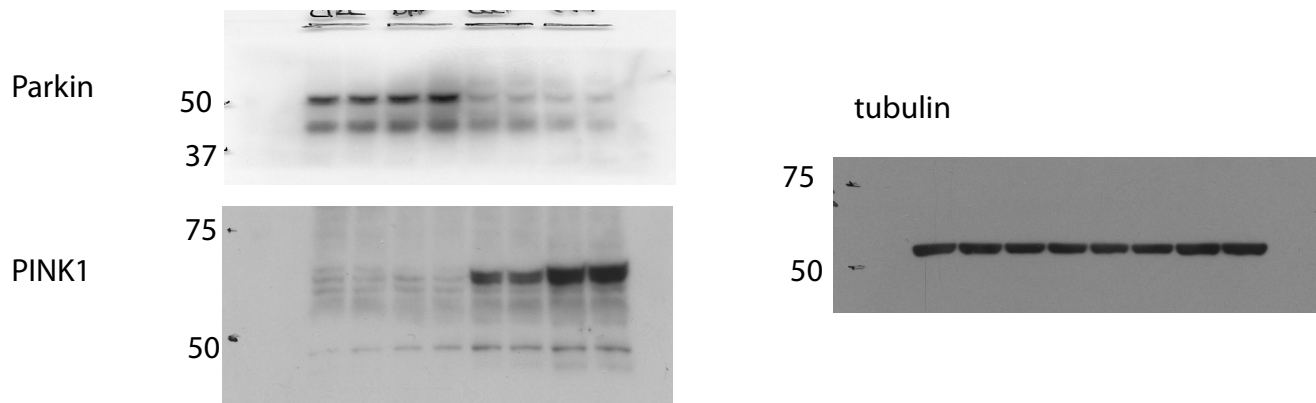

Figure 4C.

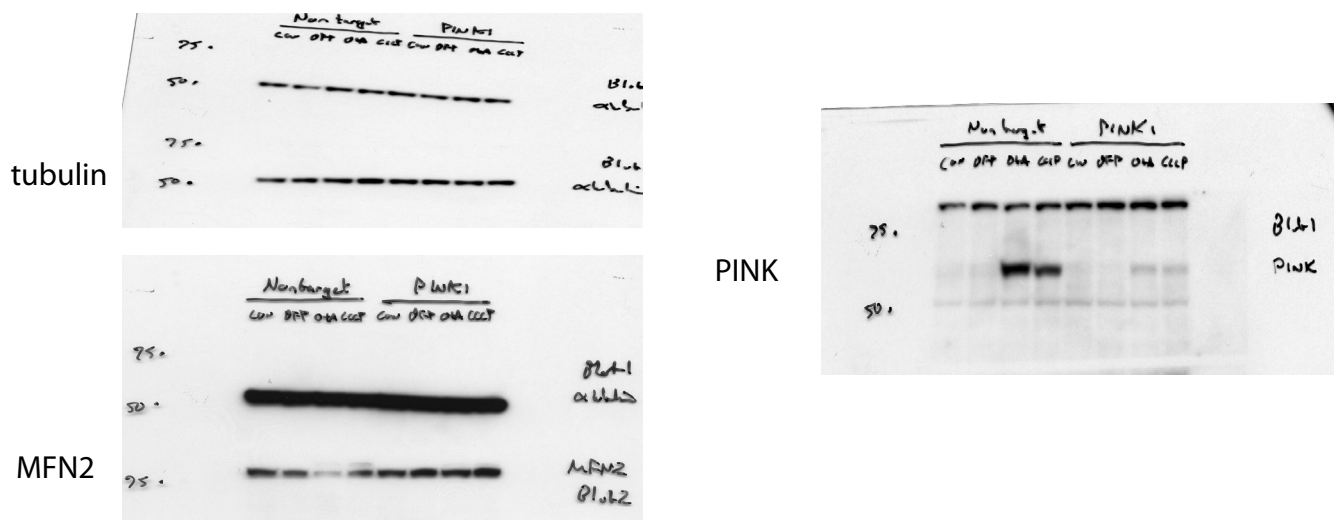

Figure 4H.

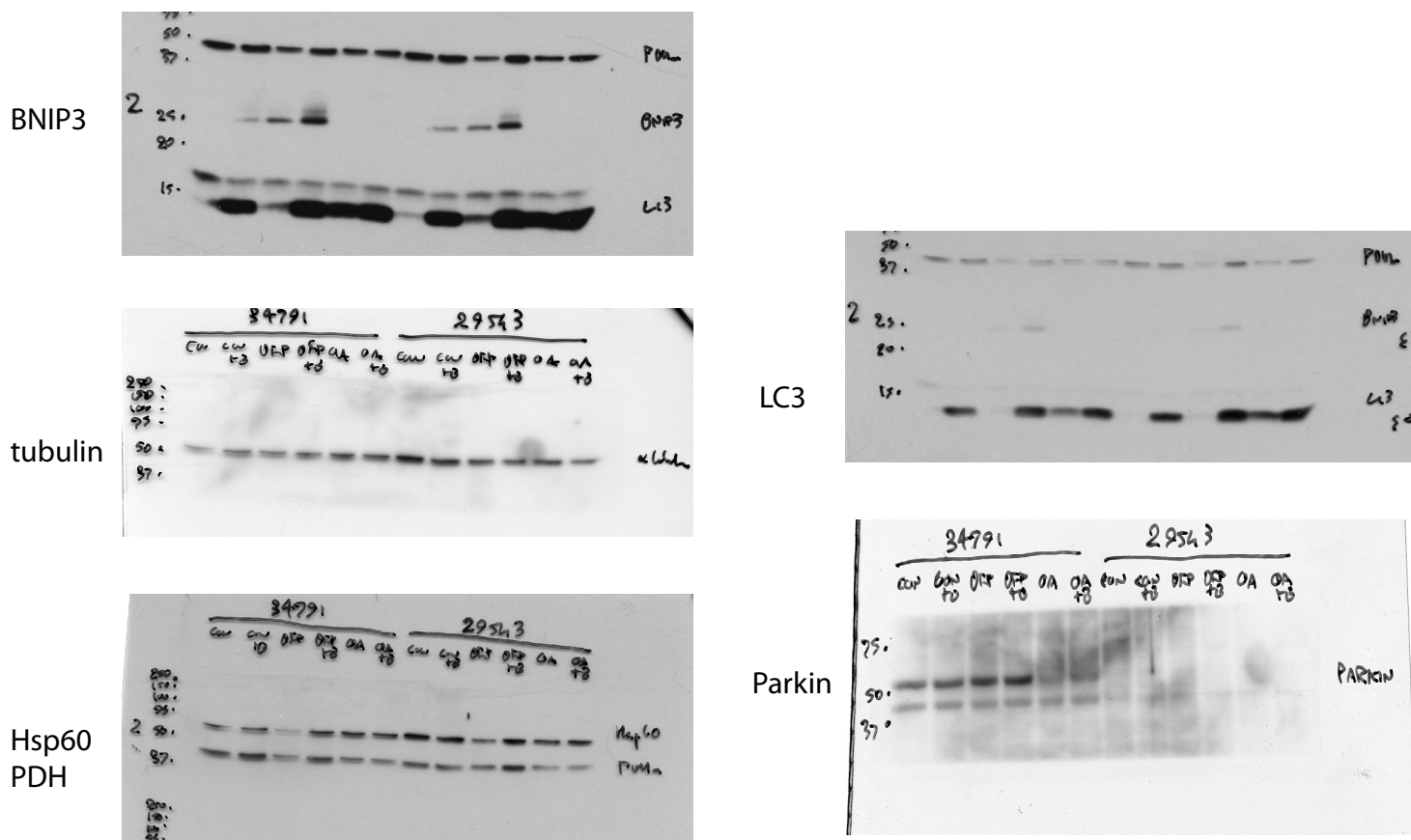

Supplement: Source data for Figure 4a [file embor2013168df4a.pdf]
